# Monitoring of *Leishmania* transmission in the postelimination phase: The potential of serological surveys

**DOI:** 10.1016/j.ijid.2024.107153

**Published:** 2024-10

**Authors:** Kristien Cloots, Om Prakash Singh, Abhishek Kumar Singh, Tulika Kumari Rai, Vishwa Deepak Tiwari, Aziza Neyaz, Sundaram Pandey, Vivek Kumar Scholar, Paritosh Malaviya, Epco Hasker, Shyam Sundar

**Affiliations:** 1 Department of Public Health, Institute of Tropical Medicine, Antwerp, Belgium; 2 Department of Biochemistry, Institute of Sciences, Banaras Hindu University, Varanasi, India; 3 Department of Medicine, Institute of Medical Sciences, Banaras Hindu University, Varanasi, India

**Keywords:** Visceral leishmaniasis, Post-elimination surveillance, Serosurveillance, Monitoring of infection, India

## Abstract

**Objectives:**

Monitoring of *Leishmania* transmission is considered a strategic priority for sustaining elimination of visceral leishmaniasis as a public health problem in the Indian subcontinent. The objective of this study was to evaluate whether serological surveys can distinguish between communities with and without *Leishmania* transmission, and to assess which serological marker performs best.

**Methods:**

Seven villages were selected from Bihar and Uttar Pradesh state, India, and categorized as either currently endemic (CE), previously endemic (PE) or nonendemic (NE). Blood samples were analyzed with the rK39 RDT, direct agglutination test (DAT), and rK39 ELISA.

**Results:**

Contrary to the rK39 RDT and DAT, the rK39 ELISA showed a significant difference between all three categories of endemicity, with a seroprevalence of 5.21% in CE villages, 1.55% in PE villages, and 0.13% in NE villages. Even when only looking at the seroprevalence among children aged <10 years, the rK39 ELISA was still able to differentiate between villages with and without ongoing transmission.

**Conclusion:**

Our findings suggest the rK39 ELISA to be the most promising marker for monitoring of *Leishmania* transmission. Further validation is required, and practical, context-adapted recommendations need to be formulated in order to guide policymakers toward meaningful and sustainable surveillance strategies in the post-elimination phase.

## Introduction

The Indian subcontinent is on the verge of eliminating visceral leishmaniasis (VL) as a public health problem. However, without **appropriate surveillance** to allow for timely actions, the health gains of achieving elimination may quickly be lost again. At present, surveillance is mainly based on monitoring the number of patients reported with VL. Going forward, however, reported cases might not be the most sensitive marker to identify resurgence early on. Although underreporting of the disease currently no longer seems to be a major issue [1], it is likely to increase again in the postelimination phase, when cases are few, and awareness within the communities and among the health care workers will be down. In addition, the vast majority of infected individuals will never develop disease; clinical cases therefore only represent the tip of the iceberg with regard to circulation of the parasite. An alternative marker is needed.

**Monitoring of transmission**, that is, monitoring the trend in infection rates, was formulated as one of the strategic priorities for accelerating and sustaining VL elimination in the Indian subcontinent [2]. Large-scale surveys measuring the prevalence of pathogen-specific antigens or antibodies in the general population already form the cornerstone of surveillance for a wide variety of communicable diseases, including vaccine-preventable diseases, emerging infectious diseases as well as other Neglected Tropical Diseases (NTDs) in an elimination setting such as lymphatic filariasis and schistosomiasis [3,4]. In addition, the NTD Roadmap 2021-2030, a high-level World Health Organization coordinated document aiming to guide the global NTD strategy in the current decade [5], emphasizes the need for integrated approaches to ensure sustainability in the long run. The introduction of surveys targeting multiple pathogens could provide a much-needed tool to help achieve this objective.

At present, however, there is **no gold standard marker** to identify *Leishmania* infection in an individual. Several serological tests do exist, but they have only been validated for diagnostic purposes in combination with VL-suggestive clinical symptoms [[6], [7], [8], [9], [10], [11]]. Nonetheless, we hypothesized that at population level, serological tests have the potential to differentiate communities with ongoing transmission from communities without. The **aim of this study** was therefore to evaluate whether serological surveys can distinguish between communities with and communities without *Leishmania (donovani)* transmission in an Indian setting, and to assess which of the markers evaluated performs best.

## Methods

### Study sites

**Figure 1:**
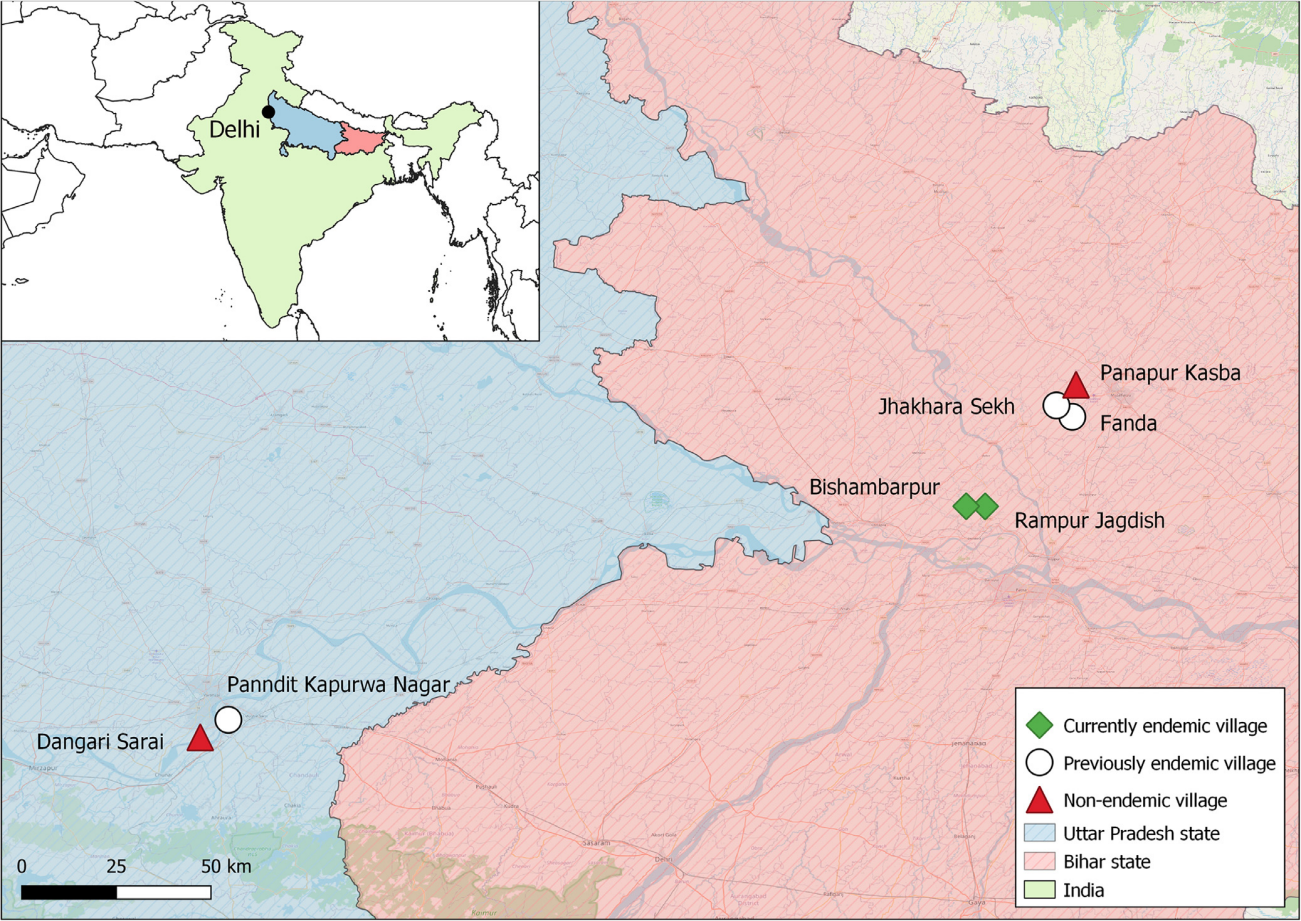
Location of study sites.

VL case data on village level were collected through the Kala-azar Management Information System database, the TMRC Health and Demographic Surveillance System data base as well as through the Public Health Center data base for the VL endemic states of Bihar and Uttar Pradesh, India. In addition, the literature was searched for any VL case reports from candidate villages before the final selection. In case of discrepancies between the different datasets, patients recorded through either source were considered a reported case. Based on the number of reported VL cases in the last 25 years, villages were classified as nonendemic (NE: no VL cases reported), previously endemic (PE: VL cases were reported previously but no more since 4 years), or currently endemic (CE: continuous VL cases in the last 4 years). From each category, two to three villages were selected based on additional criteria such as accessibility and willingness of local health authorities to collaborate, aiming at a total sample size of 15,000 (5000 per category). Figure 1 illustrates the geographical location of the seven selected study villages. VL and post-kala azar dermal leishmaniasis (PKDL; an infectious dermal sequel) case numbers reported from each respective village for the period 2005-2022 are provided in Table 1. Cases reported prior to 2005 were not included in this table, though were only relevant for a single village, that is, Panndit Kapurwa Nagar, which reported a VL outbreak in 1995 [12].

**Table 1:** VL cases reported from the selected villages (2005-2022).

State	District	Village	Disease	2005	2006	2007	2008	2009	2010	2011	2012	2013	2014	2015	2016	2017	2018	2019	2020	2021	2022	Population	Status
																		Study period					
Bihar	Saran	Rampur Jagdish	VL	0	0	0	0	1	1	1	1	0	0	1	4	11	6	0	0	0	0	4054	CE
			PKDL	0	0	0	0	0	0	0	0	0	0	0	0	0	2	0	0	0	0		
Bihar	Saran	Bishambarpur	VL	0	0	0	0	0	0	0	1	1	1	1	4	16	11	2	3	0	0	4024	CE
			PKDL	0	0	0	0	0	0	0	0	0	0	0	0	0	0	1	1	0	0		
Bihar	Muzaffarpur	Jhakara Sekh	VL	2	5	5	2	6	4	1	2	1	1	0	0	0	0	0	0	0	0	3092	PE
			PKDL	0	0	0	0	0	0	0	0	0	0	0	0	0	0	1	0	0	0		
Bihar	Muzaffarpur	Fanda	VL	15	15	13	27	11	7	5	6	4	1	0	0	0	0	0	0	0	0	3931	PE
			PKDL	0	0	0	0	0	0	0	0	0	0	0	0	0	1	0	0	0	0		
UP	Chandauli	Panndit K. N.	VL	0	0	0	0	0	0	0	0	0	0	0	0	0	0	0	0	0	0	954	PE
			PKDL	0	0	0	0	0	0	0	0	0	0	0	0	0	0	0	0	0	0		
Bihar	Muzaffarpur	Panapur Kasba	VL	0	0	0	0	0	0	0	0	0	0	0	0	0	0	0	0	1	0	1043	NE
			PKDL	0	0	0	0	0	0	0	0	0	0	0	0	0	0	0	0	0	0		
UP	Varanasi	Dangari Sarai	VL	0	0	0	0	0	0	0	0	0	0	0	0	0	0	0	0	0	0	4922	NE
			PKDL	0	0	0	0	0	0	0	0	0	0	0	0	0	0	0	0	0	0		

Population of the village is based on the inhabitants of the respective villages at the time of data collection (2019-2020).

*CE*, currently endemic cluster; *NE*, nonendemic cluster; *Panndit K.N.*, Panndit Kapurwa Nagar; *PE*, previously endemic cluster; PKDL, post-kala azar dermal leishmaniasis; *UP*, Uttar Pradesh.

### Field procedures

Between 13/05/2019 and 29/12/2020, all households of the seven selected villages were visited. All inhabitants aged ≥2 years were asked for their consent (assent for minors ≥12 years, in combination with consent from the guardian) to participate in the study. Information on demographic parameters as well as VL-related topics was collected from the consenting participants using an open-source Android application (Open Data Kit, https://docs.getodk.org/) on mobile phones, and sent to a secured server on a daily basis. A capillary blood sample was collected through finger prick to perform an rK39 Rapid Diagnostic Test (rK39 RDT; InBios, Seattle, USA) on the spot as per the manufacturer's instructions. In addition, capillary blood was collected on a Whatman filter paper, transported to the laboratory of Banaras Hindu University, Varanasi, India, and stored at −20°C until further serological testing.

### Laboratory procedures

Two additional serological tests, that is, the direct agglutination test (DAT) and the rK39 ELISA were conducted in the laboratory facilities of Banaras Hindu University, Varanasi for the detection of antibodies against *Leishmania donovani*—the only proven endemic parasite species causing VL in India.

The **DAT** was carried out using a capillary blood sample collected on a Whatman filter paper, following the established procedure [13]. In short, capillary blood filter paper disks (5 mm) were punched and placed in a 1.5 mL microcentrifuge tube. 1000 µL of DAT-buffer (1XPBS+ Ultroser G) was then added to each tube, and was incubated overnight at 4°C, resulting in a serum dilution of 1:400. The next day, 100 µL of the prepared eluates were serially diluted in a V-shaped microplate (Greiner, 96 wells, USA), reaching dilutions of up to 1:25,600 using DAT diluent (1XPBS + Ultroser G + β-mercaptoethanol). One positive and one negative control provided by the DAT manufacturer (ITM, Belgium) were included. Then, 50 µL of freeze-dried DAT antigen (ITM, Belgium) was added to each well followed by incubation for overnight at room temperature. A cut-off titer ≥1:3200 was used to define infection as suggested by El Harith and colleagues [14], but results from different DAT cut-off values are included in Figure S1.

The **rK39 ELISA** was performed as described previously [13]. Briefly, a 96-well ELISA microplate (Nunc, Thermo Fisher USA) was coated with recombinant rK39 antigen (25 ng/well) and incubated overnight at 4°C. The ELISA plate was then washed and blocked using 1% BSA (Sigma, USA) at room temperature for 2 hours. 100 µ of eluted blood, obtained from placing a 5 mm filter paper disk dispensed into 1 mL of PBS Buffer and incubating it overnight at 4°C, was then added to each well followed by incubation at room temperature for 30 minutes. The wells were washed 4 times with PBS-Tween. A protein A-horseradish peroxidase solution (1/32,000 dilution; Bangalore Genei) in PBS containing 0.1% BSA and 1.0% Tween 20 was added to the wells, and incubated for 30 minutes at room temperature. The plate was washed 4 times in PBS-T, after which 100 µL tetramethylbenzidine (Bangalore Genei) substrate was added to each well, followed by an additional incubation for a further 15 minutes in the dark. The reaction was halted by adding 1N sulfuric acid, and optical density (OD) measurements were taken at 450 nm using a microtiter plate ELISA reader (Molecular Devices, USA). Each plate included a positive (filter paper eluate from a parasitologically confirmed VL case) and a negative control (filter paper eluate from NE healthy control). The positive control served as reference to calculate a relative value of positivity for each sample, expressed as percentage positivity (PP). PP is log transformed and was calculated as the mean of 37 NE health control samples plus three times the standard deviation in reference to the positive control (mean OD of 52 active VL equals 100 PP). Samples having a PP value of ≥18 were considered positive [15].

### Data analysis

Data cleaning, analysis, and graphical presentation were performed using R studio (version 2021.09.0). Age was categorized in age groups per 10 years, with a single age group for those aged 60 years or above. Seroprevalence was calculated as the proportion of positive results among available results. Proportions were compared using the chi-square test. 95% confidence intervals around proportions were calculated using Wilson's method. Village results were grouped by endemic category (CE, PE, NE) for the main figures. To answer our primary research question on the distinction between communities with and without transmission, endemic categories were further grouped by transmission status; villages in the CE category were assumed to have ongoing transmission, villages in the PE and NE categories were assumed to have no transmission at the time of the study.

## Results

***Coverage and participant characteristics.*** A total of 3959 households were visited in the seven villages, representing a total population of 20,325, among whom 670 children below 2 years of age who were excluded from sampling. Of the 19,655 inhabitants aged ≥2 years, 78.7% (*n* = 15,468) consented to providing a blood sample for testing. Coverage ranged between 74% and 83% on village level. Overall, coverage was significantly lower among males (70%) compared to females (88%; p < 0.001) and systematically lowest in the age group 20-29 years (68%). Median age of participants was 23 years (IQR 12-40); 47% were male. A history of VL was present in 229 (3.77%), 62 (1.12%), and 5 (0.13%) participants in PE, CE, and NE villages, respectively. Detailed village-wise characteristics of the target population and participants are provided in Table S1.

**Figure 2:**
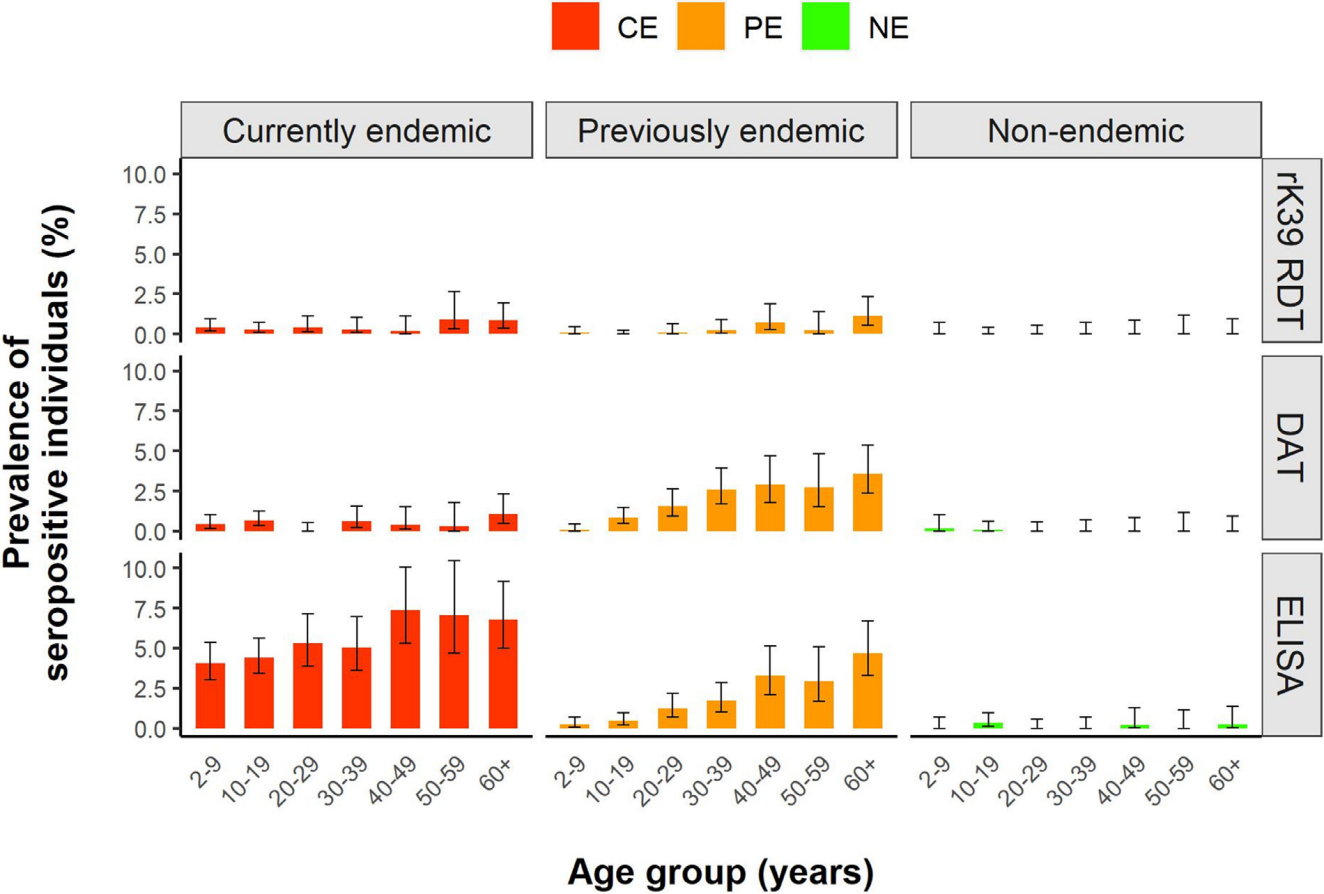
Age-wise seroprevalence results. Seroprevalence per age group per category of endemicity for the three serological tests used. *CE*, currently endemic; *NE*, nonendemic; *PE*, previously endemic.

***Age-wise seroprevalence by category of endemicity.*** A total of 15,422 rK39 RDT results were available for analysis, together with 15,132 DAT results and 15,168 rK39 ELISA results. Table 2 provides the seroprevalence results in each of the endemic categories for the three serological tests used, combining all age groups. Figure 2 provides more detailed information on seroprevalence by age group.

**Table 2:** Overall results.

Category	rK39 RDT			DAT			ELISA		
	Tested	Positive	Seroprev (95% CI)	Tested	Positive	Seroprev (95% CI)	Tested	Positive	Seroprev (95% CI)
CE	5528	23	0.42% (0.28-0.62)	5260	27	0.51% (0.35-0.75)	5262	274	5.21% (4.62-5.84)
PE	6040	16	0.26% (0.16-0.43)	6031	98	1.62% (1.34-1.98)	6065	94	1.55% (1.25-1.89)
NE	3854	0	0.00% (0.00-0.10)	3841	2	0.05% (0.01-0.19)	3841	5	0.13% (0.04-0.30)
TOTAL	15,422	39	0.25% (0.19-0.35)	15,132	127	0.84% (0.71-1.00)	15,168	373	2.46% (2.22-2.72)

Serological results per category of endemicity for the three serological tests used.

*CE*, currently endemic; *NE*, nonendemic; *PE*, previously endemic.

While no **rK39 RDT** positive results were found in the NE villages, the relatively low seroprevalence in all age groups in both the currently and PE categories did not allow to distinguish between these two.

**DAT** demonstrated a statistically significant distinction between all three categories (p < 0.001). However, the highest seroprevalence was observed in the PE villages rather than the CE villages, primarily attributable to the relatively high seroprevalence in the highest age groups in the former category, as indicated by a significant upward trend of seroprevalence with age (test for trend p < 0.001). Notably, this finding did not alter when exploring different DAT cut-off titers (Figure S1). The DAT provided two positive results in NE villages—both of them among children (<18 years) who did not report a VL history in the past and tested negative with the two other serological tests.

The **rK39 ELISA** seroprevalence results also yielded a clear distinction between the three endemic categories (p < 0.001), this time with the highest seroprevalence observed in the CE category (5.21%), followed by the PE (1.55%) and NE (0.13%) category. In terms of age-wise patterns, the NE and PE categories showed similar trends as observed with the DAT test. Specifically, there was an age-wise increase in seroprevalence in the PE category (test for trend p < 0.001) and overall few positive results in the NE category (*n* = 5; none with a history of VL, and none with a positive rK39 RDT or DAT result). Contrary to both the DAT and rK39 RDT, however, seroprevalence with the rK39 ELISA in the CE category was high across all age groups, and significantly higher than in the PE category for all except the oldest age group. Looking specifically at children <10 years (*n* = 3024) as a proxy for recent transmission, the rK39 ELISA was the only test illustrating a significantly higher seroprevalence (p < 0.001) in villages with transmission (CE; seroprevalence 4.0%) compared to the villages without transmission (PE; seroprevalence 0.2%, and NE; seroprevalence 0.0%).

Overall, 59% (23/39) of positive RDT results belonged to participants reporting a **history of VL**; for DAT and ELISA this was 54% (68/127) and 11% (42/373), respectively. Exclusion of participants with a history of VL did not alter the conclusions formulated from Figure 2.

**Figure 3:**
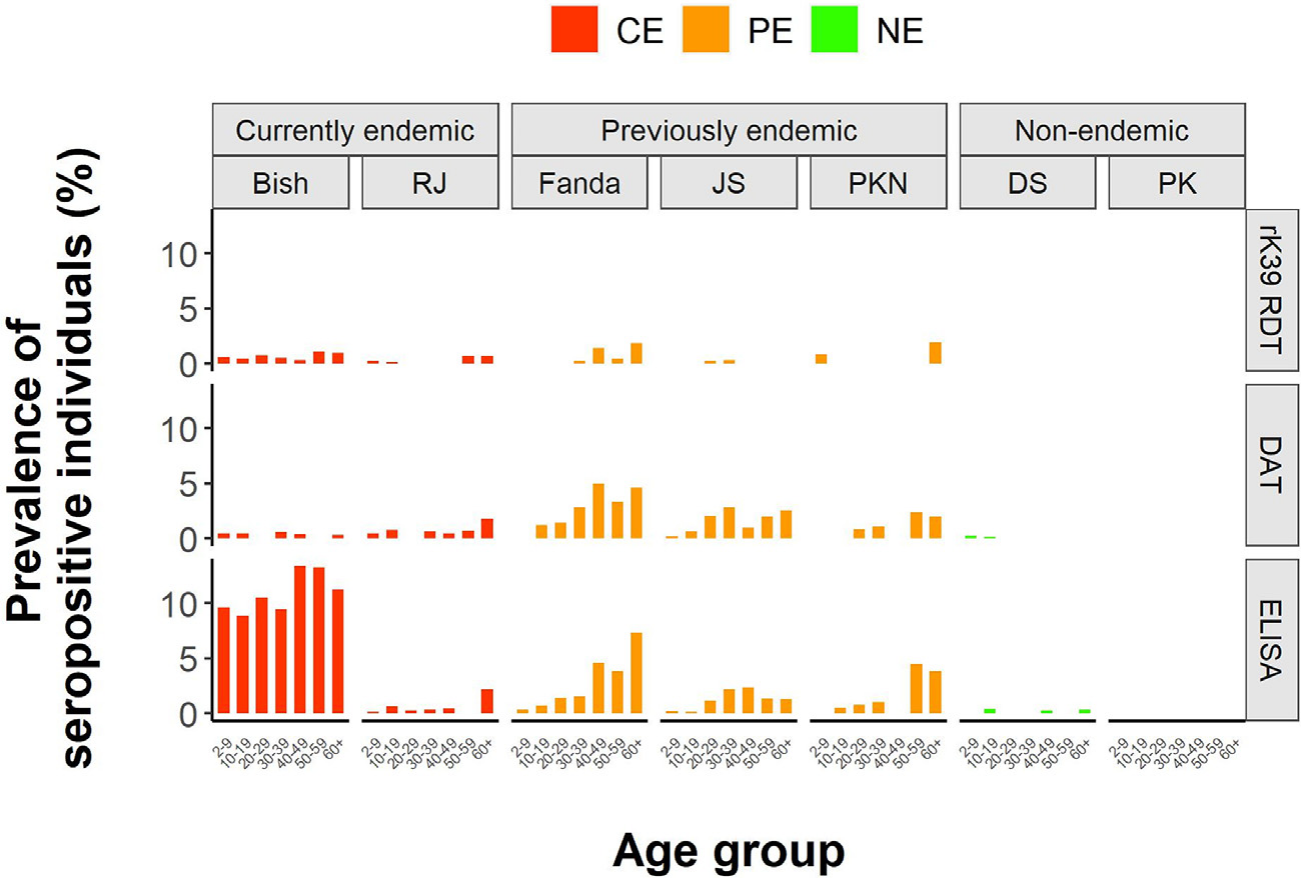
Village-wise results. Seroprevalence per age group per village for the three serological tests used. *Bish*, Bishambarpur; *CE*, currently endemic; *DS*, Dangari Sarai; *JS*, Jhakhara Sekh; *NE*, nonendemic; *PE*, previously endemic; *PK*, Panapur Kasba; *PKN*, Panndit Kapurwa Nagar; *RJ*, Rampur Jagdish.

***Age-wise results by village.*** To validate our findings for each of the different villages included in the study, we visualized serological results for each village separately in Figure 3. The graph illustrates that serological patterns for each test are relatively similar for the different villages per endemic category, except for the rK39 ELISA results among CE villages, which show a clearly higher seroprevalence in all age groups in one village (Bishambarpur) compared to the other (Rampur Jagdish). Comparing the epidemiological situation between both villages since the start of the study (Table 1), however, we observe that only the village with the high rK39 ELISA seroprevalence continued to report VL (*n* = 2) and PKDL (*n* = 3) cases since the start of the study.

## Discussion

***Summary of findings.*** Our study shows that community-wide testing with the rK39 ELISA allowed to differentiate between villages with and without ongoing *Leishmania* transmission in the Indian setting; the rK39 RDT and DAT seem less appropriate to this end. Interestingly, this statement still stands when only taking into account the seroprevalence in children below the age of 10 years, representing transmission over the last decade. Overall, these findings provide a first systematic assessment of the potential of available serological markers for monitoring of *Leishmania* transmission in the postelimination era in the Indian subcontinent, suggesting the rK39 ELISA to be the best candidate going forward.

***Implications for policymakers.*** The Regional Strategic Framework for accelerating and sustaining elimination of Kala-azar in the South-East Asia Region 2022-2026 has formulated the exploration of population-based serosurveillance to monitor trends in infection rates in the postvalidation context as one of the strategic priorities [2]. This recommendation highlights the concern that absence of reported cases does not necessarily reflect absence of transmission, but can equally well be the result of underreporting, due to low clinical awareness or flaws in the detection and reporting chain. In absence of reported cases, serological surveys could be used to validate absence of transmission. This will be especially relevant for Bangladesh, which was the first country in the Indian subcontinent to achieve certified elimination of VL as a public health problem, and in response has formulated the even more ambitious goal to interrupt *Leishmania* transmission by 2025 [16]. On the contrary, serosurveys could also be used to support the continuation or even initiation of disease control interventions if a high seroprevalence suggests transmission. A retrospective analysis of 40 years of VL case reports in Nepal illustrated that the window to act between a clear increase in caseload and a full-blown outbreak is relatively short [17]; serological surveys could provide an independent marker to pick up potential resurgence early on.

***Comparison with existing literature***. To our best knowledge, this is the first study to systematically explore the potential of serosurveillance for monitoring of *Leishmania* transmission. Nonetheless, many other studies have used serological markers to identify asymptomatic *Leishmania* infected individuals in specific communities. Most of these studies were carried out in highly VL endemic areas, while data from previously or non-endemic areas are scarce. Overall, the seroprevalence results obtained in this study seem to be lower than those generally found in the literature from the Indian subcontinent [[18], [19], [20],^15^,[21], [22], [23]]. However, our findings should be interpreted in light of the systematic decrease in VL cases and likely also transmission over time as a result of the elimination efforts in the Indian subcontinent [20]. Overall, there is important variation in *Leishmania* seroprevalence results in the literature. As agreement between different markers of infection is generally low, part of these differences can be explained by the use of different markers in different studies. The rK39 ELISA has been suggested to be more sensitive than DAT for identifying early infection, though little is known about the exact dynamics of the different serological markers [24]. Other factors contributing to this heterogeneity likely include differences in intensity of transmission at the time of the study as well as in preceding years, the population targeted for sampling (e.g., only household contacts of VL cases versus whole communities, with or without exclusion of ex-VL cases), and the cut-off value used to define seropositivity when using (semi-)quantitative markers.

***Study limitations.*** This study comes with several limitations. First, other markers for infection exist which do not rely on antibody titers, including molecular markers (e.g., PCR), cellular markers (e.g., Leishmanin Skin Test), and antigen-based markers (e.g., urinary antigen); these were not assessed in the current study as they were deemed less feasible for large-scale use in their current formats. Second, attribution of a clinical case to a village was based on place of residence of the patient at the time of diagnosis, without taking into account the possibility of an imported infection. Identifying the place of infection is extremely challenging for patients residing in a larger area considered endemic—as was the case in this study will all villages being located in the two VL endemic states of Bihar and UP. This is especially relevant in view of the village labeled as NE during the study which reported a single VL patient in the year after study completion (see Table 1). Without additional cases appearing in the following years, however, it seems likely that such sporadic cases are either the result of an infection that took place elsewhere and/or of an infection that took place long ago but has only become clinically apparent due to a weakening immune system.

Another study limitation was that endemic categories were based on the number of clinical VL cases reported in the 25 years prior to the study period, without additional activities such as active case detection or retrospective case confirmation to validate their status. It is possible therefore that some cases remained unreported in previous years, potentially leading to misclassification. It should be highlighted that the two solitary PKDL cases reported in PE villages during and right before the study period (see Table 1) do not contradict the categorization of these villages as “previously endemic,” as PKDL should be seen as a sequel occurring many years after the initial infection, and usually preceded by a VL episode, though no further information on clinical history was available from these patients.

Important to note is that there was a clear difference in rK39 ELISA seroprevalence results between the two villages with ongoing transmission (Bishambarpur and Rampur Jagdish). Taking into account the fact that Rampur Jagdish did not report any more clinical VL cases in the 4 years since the start of the study period, we could argue that transmission in this village had effectively already been halted at the time of the study, and that it should therefore have been considered a PE village rather than a CE village. This poststudy finding seems to support the claims made on the meaning of village-wide rK39 ELISA seroprevalence. Nonetheless, it also emphasizes the need for further validation of the results, since they mostly rely on the findings from a single village (Bishambarpur).

***Going forward***, context-specific sample sizes and cut-off values will need to be explored, depending on the question to be answered. A similar approach to the Transmission Assessment Surveys carried out for lymphatic filariasis could be considered, in which a predefined sample size with an associated threshold seroprevalence guides (dis)continuation of public health actions. The sample size and threshold values will also depend on the population targeted for sampling, and the expected seroprevalence in this group in case of presence or absence of transmission. Children could provide a specific population of interest in this regard, as the detection of antibodies in this group indicates continued transmission in the years since their birth, and possibly a need to trigger public health action [25]. In this study, the rK39 ELISA was able to distinguish between villages with and without ongoing transmission based on seroprevalence of children <10 years alone (seroprevalence 4.0% and 0.17%, respectively, p < 0.001), suggesting that also for monitoring of *Leishmania* transmission children could be an interesting target group.

While further exploring the rK39 ELISA as a tool for monitoring of *Leishmania* transmission, the quest for better—serological or nonserological—markers should continue unabated. In addition, it will be important to explore opportunities for integration with other diseases, as this will be a crucial step toward sustainability in the long run [5,2,26]. Integrated serosurveillance has already been used in different settings and for different pathogens, but context-adapted options will need to be formulated specifically for the Indian subcontinent [25,27,28,29]. Especially other NTDs in an elimination setting, such as lymphatic filariasis and leprosy, might provide meaningful candidates for integrated approaches, as they can be assumed to face similar challenges with regard to sustainability. Recent developments such as multiplex platforms could further optimize the efficiency of much-needed platforms for integrated surveillance [28,30].

## Conclusions

Integrated serological surveys have been identified as a priority strategy to enhance sustainability of surveillance for NTDs including VL in the post-elimination phase in the Indian subcontinent [2]. This study suggests the rK39 ELISA to be the most promising candidate available at present for VL. Further validation is required, and practical, context-adapted recommendations need to be formulated in order to guide policymakers toward meaningful and sustainable surveillance strategies in the postelimination phase.

## Data sharing statement

The data supporting the findings of this study are retained at Banaras Hindu University, Varanasi (India), and will not be made openly accessible due to ethical and privacy concerns. Data can however be made available after approval of a motivated and written request to the authors (opsinghbiochem@bhu.ac.in).

## Ethical approval

The study was conducted according to the guidelines of the Declaration of Helsinki. Ethical approval was obtained from the relevant committees in India (Ethics Committee of Banaras Hindu University in Varanasi) and Belgium (Institutional Review Board of the Institute of Tropical Medicine, Ethics Committee of the University of Antwerp Hospital in Antwerp).

## Declarations of competing interest

The authors declare that they have no known competing financial interests or personal relationships that could have appeared to influence the work reported in this article.

## Appendix Supplementary materials

Image, application 1

## References

[1] Singh SP, Reddy DC, Rai M, Sundar S. (2006). Serious underreporting of visceral leishmaniasis through passive case reporting in Bihar, India. Trop Med Int Health.

[2] World Health Organization (2022). . Regional strategic framework for accelerating and sustaining elimination of kala-azar in the South-East Asia region. 2022-2026.

[3] World Health Organization, Geneva (2011).

[4] World Health Organization (2016). Towards eliminating lymphatic filariasis: progress in the South-East Asia Region.

[5] World Health Organization (2020).

[6] Bern C, Hightower AW, Chowdhury R, Ali M, Amann J, Wagatsuma Y (2005). Risk factors for kala-azar in Bangladesh. Emerg Infect Dis.

[7] Boelaert M, Rijal S, Regmi S, Singh R, Karki B, Jacquet D (2004). A comparative study of the effectiveness of diagnostic tests for visceral leishmaniasis. Am J Trop Med Hyg.

[8] Boelaert M, Verdonck K, Menten J, Sunyoto T, van Griensven J, Chappuis F (2014). Rapid tests for the diagnosis of visceral leishmaniasis in patients with suspected disease. Cochrane Database Syst Rev.

[9] Chappuis F, Rijal S, Soto A, Menten J, Boelaert M. (2006). A meta-analysis of the diagnostic performance of the direct agglutination test and rK39 dipstick for visceral leishmaniasis. BMJ (Clinical Res Ed).

[10] Kassa M, Abdellati S, Cnops L, Bremer Hinckel BC, Yeshanew A, Hailemichael W (2020). Diagnostic accuracy of direct agglutination test, rK39 ELISA and six rapid diagnostic tests among visceral leishmaniasis patients with and without HIV coinfection in Ethiopia. PLoS Negl Trop Dis.

[11] Salam MA, Huda MM, Khan MGM, Shomik MS, Mondal D. (2021). Evidence-based diagnostic algorithm for visceral leishmaniasis in Bangladesh. Parasitol Int.

[12] Kumar R, Kumar P, Chowdhary RK, Pai K, Mishra CP, Kumar K (1999). Kala-azar epidemic in Varanasi district, India. Bull World Health Organ.

[13] Chakravarty J, Hasker E, Kansal S, Singh OP, Malaviya P, Singh AK (2019). Determinants for progression from asymptomatic infection to symptomatic visceral leishmaniasis: a cohort study. PLoS Negl Trop Dis.

[14] Harith AE, Kolk AH, Kager PA, Leeuwenburg J, Muigai R, Kiugu S (1986). A simple and economical direct agglutination test for serodiagnosis and sero-epidemiological studies of visceral leishmaniasis. Trans R Soc Trop Med Hyg.

[15] Hasker E, Kansal S, Malaviya P, Gidwani K, Picado A, Singh RP (2013). Latent infection with *Leishmania donovani* in highly endemic villages in Bihar, India. PLoS Negl Trop Dis.

[16] National Kala-azar Elimination Programme (2023). . Directorate General of Health Services, Ministry of Health and Family Welfare, Government of the People’s Republic of Bangladesh. Costed National Strategic Plan for Kala-azar Elimination in Bangladesh 2020-2030.

[17] Pandey K, Dumre SP, Shah Y, Acharya BK, Khanal L, Pyakurel UR (2023). Forty years (1980-2019) of visceral leishmaniasis in Nepal: trends and elimination challenges. Trans R Soc Trop Med Hyg.

[18] Basnyat S, Banjara MR, Ghimire P, Matlashewski G, Singh A. (2021). Seropositivity of visceral leishmaniasis on people of VL endemic three districts of Nepal. Parasitol Int.

[19] Basnyat S, Banjara MR, Ghimire P, Matlashewski G, Singh A. (2022). Investigation of visceral LeishmaniasisTransmission in selected districts of Nepal. J Nepal Health Res Counc.

[20] Cloots K, Uranw S, Ostyn B, Bhattarai NR, Le Rutte E, Khanal B (2020). Impact of the visceral leishmaniasis elimination initiative on *Leishmania donovani* transmission in Nepal: a 10-year repeat survey. Lancet Glob Health.

[21] Ostyn B, Gidwani K, Khanal B, Picado A, Chappuis F, Singh SP (2011). Incidence of symptomatic and asymptomatic *Leishmania donovani* infections in high-endemic foci in India and Nepal: a prospective study. PLoS Negl Trop Dis.

[22] Rijal S, Uranw S, Chappuis F, Picado A, Khanal B, Paudel IS (2010). Epidemiology of *Leishmania donovani* infection in high-transmission foci in Nepal. Trop Med Int Health.

[23] Singh SP, Picado A, Boelaert M, Gidwani K, Andersen EW, Ostyn B (2010). The epidemiology of *Leishmania donovani* infection in high transmission foci in India. Trop Med Int Health.

[24] Zijlstra EE, Daifalla NS, Kager PA, Khalil EA, El-Hassan AM, Reed SG (1998). rK39 enzyme-linked immunosorbent assay for diagnosis of *Leishmania donovani* infection. Clin Diagn Lab Immunol.

[25] Hatherell HA, Simpson H, Baggaley RF, Hollingsworth TD, Pullan RL. (2021). Sustainable surveillance of neglected tropical diseases for the post-elimination era. Clin Infect Dis.

[26] World Health Organisation, Regional Office for South-East Asia, New Delhi, India. Meeting of programme managers and the RTAG for the kala-azar elimination programme. 2022. Available from: https://www.gavi.org/vaccineswork/cutaneous-leishmaniasis-spikes-pakistan-treatment-stock-outs-hit-northwestern. [acessed 24 January 2024].

[27] Al Abaidani I, Al-Abri S, Shaban M, Ghugey SL, Al Kathery S, Al-Mashikhi K (2016). Decline in transmission of *Schistosomiasis mansoni* in Oman. Infect Dis Poverty.

[28] Fornace KM, Senyonjo L, Martin DL, Gwyn S, Schmidt E, Agyemang D (2022). Characterising spatial patterns of neglected tropical disease transmission using integrated sero-surveillance in Northern Ghana. PLoS Negl Trop Dis.

[29] Saboyá-Díaz MI, Castellanos LG, Morice A, Ade MP, Rey-Benito G, Cooley GM (2023). Lessons learned from the implementation of integrated serosurveillance of communicable diseases in the Americas. Rev Panam Salud Publica.

[30] Chan YL, Patterson CL, Priest JW, Stresman G, William T, Chua TH (2022). Assessing seroprevalence and associated risk factors for multiple infectious diseases in Sabah, Malaysia using serological multiplex bead assays. Front Public Health.

