# Supplementary material for: Monitoring of Leishmania transmission in the postelimination phase: The potential of serological surveys
Source: Int J Infect Dis. 2024 Oct;147:None. doi: 10.1016/j.ijid.2024.107153 (PMC11442319; doi:10.1016/j.ijid.2024.107153)

**Supplementary Materials**

**Table S1: Target population and participant characteristics per study cluster. Characteristics of the target population (aged 2 years and above) and the participants (providing a blood sample) per cluster. Coverage was calculated as the number of participants among the target population. Me = median; IQR = interquartile range; CE = currently endemic; PE = previously endemic; NE = non-endemic.**

| **Cluster** |  |  |  | **Rampur Jagdish** | **Bishambarpur** | **Jhakara Sekh** | **Fanda** | **Panndit Kapurwa Nagar** | **Panapur Kasba** | **Dangari Sarai** | **OVERALL** |
| --- | --- | --- | --- | --- | --- | --- | --- | --- | --- | --- | --- |
| **Status** | | |  | **CE** | **CE** | **PE** | **PE** | **PE** | **NE** | **NE** |  |
| **Target population** | | |  |  |  |  |  |  |  |  |  |
|  | Block | |  | Dariapur | Dariapur | Marwan | Marwan | Niyamatabad | Kanti | Kashi Vidyapeeth |  |
|  | District | |  | Saran | Saran | Muzaffarpur | Muzaffarpur | Chandauli | Muzaffarpur | Varanasi |  |
|  | State | |  | Bihar | Bihar | Bihar | Bihar | UP | Bihar | UP |  |
|  | No of inhabitants | |  | 3748 | 3619 | 2949 | 3780 | 849 | 1004 | 3706 | **19655** |
|  | No of HH | |  | 730 | 761 | 600 | 802 | 153 | 210 | 703 | **3959** |
|  | HH members (Me(IQR)) | |  | 5 (4-6) | 5 (4-6) | 5 (4-6) | 5 (4-6) | 5 (4-7) | 5 (4-6) | 5 (4-6) | **5 (4-6)** |
|  | Age in years (Me(IQR)) | |  | 20 (10-37) | 24 (12-40) | 22 (11-38) | 23 (12-40) | 22 (13-36) | 26 (15-45) | 26 (15-40) | **23 (12-40)** |
|  | Age distribution | |  |  |  |  |  |  |  |  |  |
|  |  | 2-9 |  | 821 (22%) | 616 (17%) | 607 (21%) | 689 (18%) | 137 (16%) | 144 (14%) | 482 (13%) | **3496 (18%)** |
|  |  | 10-19 |  | 994 (27%) | 914 (25%) | 715 (24%) | 915 (24%) | 221 (26%) | 223 (22%) | 865 (23%) | **4847 (25%)** |
|  |  | 20-29 |  | 598 (16%) | 607 (17%) | 497 (17%) | 632 (17%) | 180 (21%) | 179 (18%) | 752 (20%) | **3445 (18%)** |
|  |  | 30-39 |  | 486 (13%) | 510 (14%) | 420 (14%) | 544 (14%) | 123 (14%) | 124 (12%) | 569 (15%) | **2776 (14%)** |
|  |  | 40-49 |  | 333 (9%) | 377 (10%) | 274 (9%) | 367 (10%) | 74 (9%) | 121 (12%) | 399 (11%) | **1945 (10%)** |
|  |  | 50-59 |  | 199 (5%) | 238 (7%) | 169 (6%) | 255 (7%) | 54 (6%) | 86 (9%) | 296 (8%) | **1297 (7%)** |
|  |  | 60+ |  | 317 (8%) | 357 (10%) | 267 (9%) | 378 (10%) | 60 (7%) | 127 (13%) | 343 (9%) | **1849 (9%)** |
|  | No (%) male | |  | 1976 (53%) | 1917 (53%) | 1558 (53%) | 2029 (54%) | 437 (51%) | 526 (52%) | 1906 (51%) | **10349 (53%)** |
|  | No (%) VL history | |  | 35 (0.9%) | 35 (1.0%) | 82 (2.8%) | 164 (4.3%) | 18 (2.1%) | 5 (0.5%) | 0 (0.0%) | **339 (1.7%)** |
| **Participants** | | |  |  |  |  |  |  |  |  |  |
|  | Age in years (Me(IQR)) | | | 18 (10-38) | 24 (12-42) | 20 (10-39) | 22 (11-40) | 22 (12-37) | 28 (14-48) | 25 (15-42) | **23 (12-40)** |
|  | No (%) VL history | | | 31 (0.8%) | 31 (0.9%) | 73 (2.5%) | 138 (3.7%) | 18 (2.1%) | 5 (0.5%) | 0 (0.0%) | **296 (1.5%)** |
|  | Coverage |  |  |  |  |  |  |  |  |  |  |
|  |  | Overall (n (%)) | | 2773 (74%) | 2756 (76%) | 2419 (82%) | 2983 (79%) | 679 (80%) | 788 (78%) | 3070 (83%) | **15468 (79%)** |
|  |  | By age group (n (%)) | |  |  |  |  |  |  |  |  |
|  |  |  | 2-9 | 678 (83%) | 524 (85%) | 549 (90%) | 615 (89%) | 118 (86%) | 120 (83%) | 420 (87%) | **3024 (86%)** |
|  |  |  | 10-19 | 755 (76%) | 691 (76%) | 598 (84%) | 730 (80%) | 185 (84%) | 178 (80%) | 741 (86%) | **3878 (80%)** |
|  |  |  | 20-29 | 376 (63%) | 386 (64%) | 347 (70%) | 426 (67%) | 126 (70%) | 111 (62%) | 575 (76%) | **2347 (68%)** |
|  |  |  | 30-39 | 319 (66%) | 373 (73%) | 326 (78%) | 394 (72%) | 96 (78%) | 95 (77%) | 438 (77%) | **2041 (74%)** |
|  |  |  | 40-49 | 222 (67%) | 281 (75%) | 210 (77%) | 281 (77%) | 57 (77%) | 97 (80%) | 348 (87%) | **1496 (77%)** |
|  |  |  | 50-59 | 146 (73%) | 184 (77%) | 150 (89%) | 210 (82%) | 45 (83%) | 74 (86%) | 252 (85%) | **1061 (82%)** |
|  |  |  | 60+ | 277 (87%) | 317 (89%) | 239 (90%) | 327 (87%) | 52 (87%) | 113 (89%) | 296 (86%) | **1621 (88%)** |
|  |  | By sex (n (%)) | |  |  |  |  |  |  |  |  |
|  |  |  | Male | 1220 (62%) | 1281 (67%) | 1153 (74%) | 1401 (69%) | 311 (71%) | 385 (73%) | 1487 (78%) | **7238 (70%)** |
|  |  |  | Female | 1553 (88%) | 1475 (87%) | 1266 (91%) | 1582 (90%) | 368 (89%) | 403 (84%) | 1583 (88%) | **8230 (88%)** |

**Figure_S1: Seroprevalence per age group per endemic status for different cut-off values of DAT.**


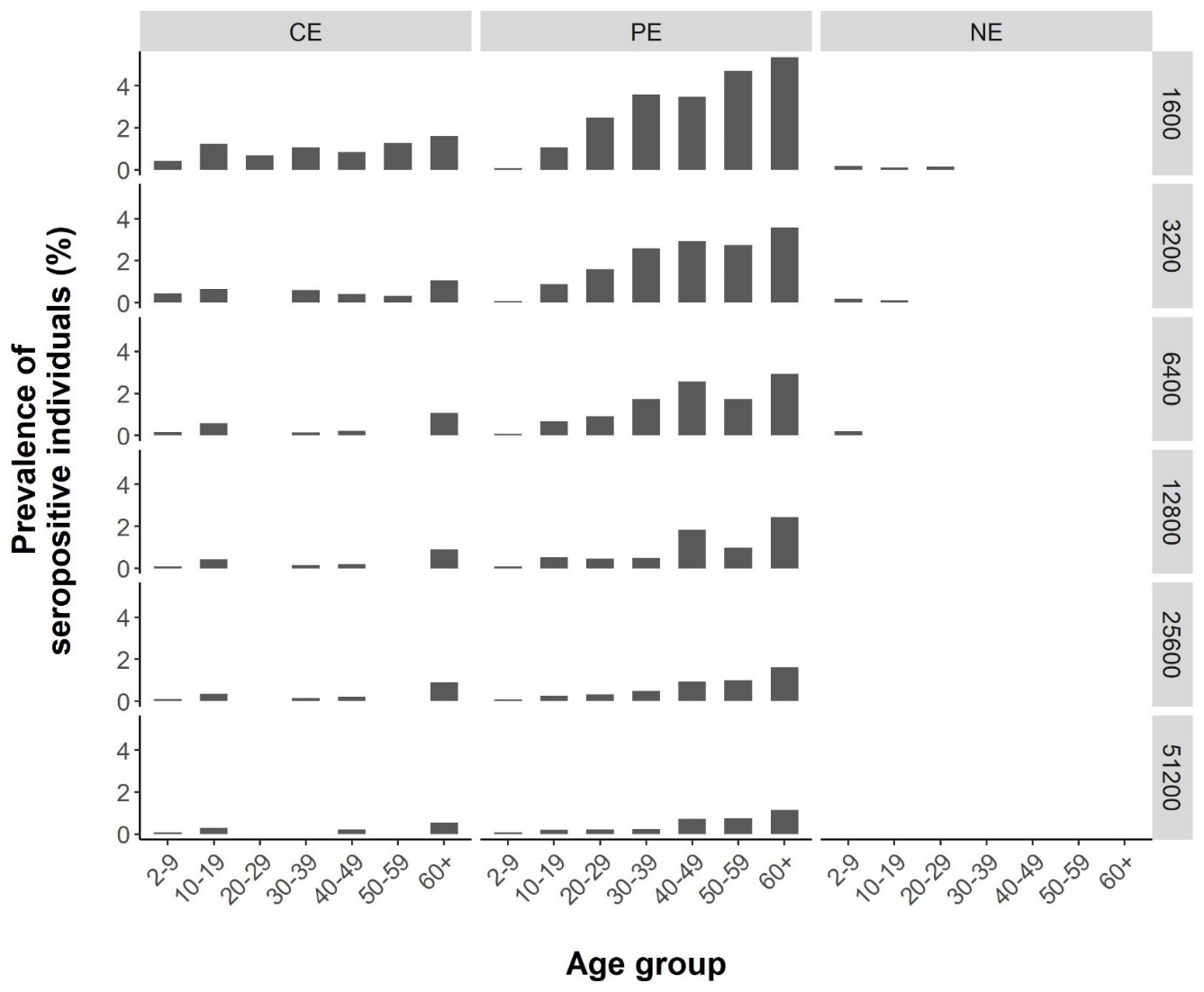

Supplement: Supplementary file 1 [file mmc1.docx]
